# Mechanical Efficiency and Injury Risk in Leg Kicks Across Combat Sports: A Narrative Review of Stance, Hip Rotation, and Striking Surface Effects

**DOI:** 10.3390/healthcare14040430

**Published:** 2026-02-09

**Authors:** Soheil Sabri Razm, Kalenia Márquez-Flórez, Lucio Caprioli, Cristian Romagnoli, Saeid Edriss, Ida Cariati, Roberto Bonanni, Francesca Campoli, Virginia Tancredi, Elvira Padua, Giuseppe Annino

**Affiliations:** 1 Human Performance Laboratory, Centre of Space Bio-Medicine, Department of Medicine Systems, University of Rome Tor Vergata, I-00133 Rome, Italy; soheil.sabrirazm@students.uniroma2.eu (S.S.R.); giuseppe.annino@uniroma2.it (G.A.); 2 Arts et Métiers Institute of Technology, EPF Engineering School, Université Sorbonne Paris Nord, IBHGC–Institut de Biomécanique HumaineGeorges Charpak, F-75013 Paris, France; 3 Sports Engineering Laboratory, Department of Industrial Engineering, University of Rome Tor Vergata, I-00133 Rome, Italy; saeid.edriss@alumni.uniroma2.eu; 4 Department of Human Science and Promotion of Quality of Life, San Raffaele Rome University, I-00166 Rome, Italy; roberto.bonanni@uniroma5.it (R.B.); francesca.campoli@uniroma5.it (F.C.); elvira.padua@uniroma5.it (E.P.); 5 Department of Systems Medicine, “Tor Vergata” University of Rome, Via Montpellier 1, I-00133 Rome, Italy; ida.cariati@uniroma2.it (I.C.); tancredi@uniroma2.it (V.T.)

**Keywords:** kick biomechanics, taekwondo, stance stability, lower limb injuries, injury prevention, performance optimization

## Abstract

**Highlights:**

**What are the main findings?**

- Pivot optimizes hip rotation and power but increases rotational stress on the knee and ankle of the supporting leg (risk of ACL and sprains).
- Contact with the tibia (shin) maximizes momentum but is associated with a high risk of tibial contusions and stress fractures (typical of Muay Thai/MMA).
- Contact with the instep promotes speed and scoring but exposes the foot and ankle to sprains and fractures.

**What are the implications of the main findings?**

- The same technical choices that maximize performance (impact force and speed) create specific vulnerabilities to injury.
- Results highlight a performance-safety continuum.

**Abstract:**

Leg kicks are fundamental techniques in combat sports based on a proximal-distal sequence involving several factors that can affect mechanical efficiency and injury risk. However, there is a lack of comprehensive reviews that integrate biomechanical and epidemiological evidence on injuries in an interdisciplinary context. **Background/Objectives**: This narrative review synthesizes current evidence to explore the relationship between mechanical efficiency and injury risk in kick-based combat sports. **Methods**: The search was conducted across Web of Science and Scopus (January 2000–March 2025) or studies investigating the biomechanics and injury risk factors associated with leg kicks in Taekwondo, Karate, Muay Thai, Kickboxing, and MMA. **Results**: Analysis of 23 studies identified three primary technical determinants of efficiency: stance mechanics, hip rotation, and striking-surface selection. High-impact force is consistently associated with a pivoted support leg stance and proximal-to-distal coordination. However, these same mechanics create specific “load concentrations” that align with documented injury profiles: pivoted stances increase rotational stress on the support leg knee (ACL/meniscal strain), while striking-surface choice (shin vs. instep) dictates the trade-off between tibial stress and metatarsal/ankle trauma. **Conclusions**: This review proposes an Integrated mechanical efficiency–injury model that suggests that performance optimization and injury awareness are two sides of the same biomechanical process. Future research should fill the gaps relating to the subject’s age and gender parity, as well as direct comparisons between different sports.

## 1. Introduction

Combat sports (CS) represent a variety of disciplines in which the human body is simultaneously an instrument of attack, a target, and a defense system, and in which there is often a subtle interaction between biomechanical principles and applied strategy [1,2]. In CS such as Muay Thai, Kickboxing, Mixed Martial Arts (MMA), Karate, and Taekwondo, leg kicks are fundamental techniques [3,4,5]. They serve key tactical functions, including disrupting opponents’ balance, creating openings, and accumulating damage [6]. From a biomechanical perspective, effective leg kicking relies on proximal-to-distal sequencing, with stance, hip rotation, and striking surface selection being critical determinants for force transmission and postural stability [6,7,8]. These biomechanical characteristics influence both performance outcomes and injury mechanisms across CS.

Mechanical efficiency in martial arts kicking can be conceptualized as the ability to maximize mechanical output (e.g., foot velocity, joint angular velocity, impact force, or kinetic energy) relative to the neuromuscular and metabolic effort required, while minimizing energy losses due to unnecessary muscle co-contraction or inefficient movement coordination [9,10]. In general, high mechanical efficiency is achieved through the proximal-to-distal coordination of segmental motion, producing high distal segment velocities or impact energies without excessive muscle effort or compromising posture [11,12]. This concept is crucial as a biomechanical framework to compare kicking techniques across combat sports.

However, mechanical efficiency must be interpreted in light of the sport-specific constraints. What qualifies as an “effective” kick depends on the rules, tactical objectives, and scoring or impact criteria of each combat sport, which may prioritize speed, accuracy, contact force, or repeated execution [13,14]. Moreover, the biomechanical characteristics that might enhance performance may also increase musculoskeletal loading: pivoting may strain the support knee, shin contact may increase tibial stress, and instep striking may expose the foot to fractures [15,16,17,18]. Consequently, efficient kicking performance reflects a compromise between mechanical efficiency and injury risk, underscoring the need for integrative analyses that include both performance outcomes and injury mechanisms.

Existing literature on leg kicking remains largely fragmented and discipline-specific [19,20]. While such specialization is necessary given the specific characteristics of the different combat disciplines (technical, tactical, and constraining), it limits the comparison across CS and hinders the development of unified biomechanical principles of the efficacy and injury-free kicking. Hence, performance and injury outcomes are frequently examined in isolation or within a single sporting context.

This fragmentation is evident in the focus of performance-oriented studies; studies in Taekwondo emphasize speed and scoring-related variables [5,8], whereas research in Muay Thai or MMA prioritizes impact force and damage-related outcomes [4,18]. Injury surveillance studies show a similar sport-specific pattern, documenting foot and ankle injuries in instep-dominant sports [15,21] and shin or thigh trauma in shin-based styles [4,18]. The lack of a consistent methodological framework and common outcome measures complicates evidence synthesis, hides shared common performance and risk factors, and limits the translation of findings into cross-disciplinary training or injury-prevention strategies. Thus, studies integrating biomechanical and injury evidence to evaluate how stance, hip rotation, and strike-surface collectively influence both performance and injury risk across CS are lacking in the literature.

Previous reviews on kicking have been limited to specific disciplines, such as Taekwondo performance analyses or Karate injury epidemiology, without providing synthesis findings across different striking styles. To fill this gap, this review encompasses peer-reviewed studies published between January 2000 and March 2025, focusing on CS in which leg kicks are fundamental techniques. By combining evidence from biomechanics and sports medicine, the present study provides a cross-disciplinary synthesis aimed at advancing theoretical understanding, informing athlete preparation, and supporting injury prevention in combat sports. In line with the performance–injury framework outlined above, this review assumes that performance optimization and athlete safety should never be treated as separate goals but as interdependent aspects of high-level training and competition.

## 2. Materials and Methods

### 2.1. Eligibility

The inclusion criteria for this review were the following: (i) original articles published in English; (ii) studies published between January 2000 and March 2025; (iii) the population of the study was CS athletes, regardless of sex, age, or competitive level; (iv) athletes practicing CS in which leg kicks are fundamental (e.g., Muay Thai, Kickboxing, MMA, Karate, Taekwondo, Wushu); (v) studies reporting at least one biomechanical aspect (e.g., kinematic variables such as joint angles, angular velocities, and execution time; kinetic variables such as impact force, impulse, ground reaction forces, or joint moments; electromyographic activity or assessments of postural control and stability) or injury-related outcomes associated with leg kick execution; (vi) study designs including experimental, quasi-experimental, cross-sectional, prospective, or retrospective studies that reported primary data [22]. The exclusion criteria were as follows: (i) prospective studies or interventions not focused on CS, (ii) publications that did not correspond to original research (e.g., letters to the editor, translations, notes, and book reviews), (iii) case studies with one single athlete.

### 2.2. Information Sources and Search Strategy

A comprehensive literature search was conducted in the Web of Science Core Collection and Scopus databases, which are widely recognized as reliable sources for scientific publications [23,24].

The search strategy combined keywords related to leg kicks, biomechanics, injury, and combat sports, using Boolean operators to retrieve studies focused on kicking-related outcomes. It was performed within the Title, Abstract, and Keywords fields of the records. The search string was as follows: (“Mechanical efficiency” OR “Injury risk” OR “Leg kicks” OR “Hip rotation” OR “Stance” OR “Surface-strike”) AND (“Combat Sport” OR “taekwondo” OR “martial arts” OR “karate” OR “MMA” OR “wushu” OR “muay Thai” OR “kickboxing“).

### 2.3. Study Selection and Data Extraction

All search results were exported fully recorded from Web of Science and Scopus datasets using Rayyan Software (Version 1.4.3). Duplicates across searches were systematically identified and removed using Digital Object Identifier (DOI) filtering. The remaining records were screened in three sequential stages.

(1) Title and Abstract Screening: Records clearly unrelated to combat sports, leg kicks, or biomechanics/injury outcomes (e.g., studies on Wrestling, Judo, or upper limb strikes) were excluded.
(2) Screening Groups: Each remaining record was categorized as “Included”, “Excluded”, or “Maybe”. Studies in the “Maybe” category were not clearly irrelevant but required full-text review to determine eligibility.
(3) Full-Text Screening: Full texts were retrieved for all “Included” and “Maybe” articles. Studies that did not meet the eligibility criteria or were unavailable in full text were excluded.

### 2.4. Assessment of Methodological Quality

To evaluate the reliability of the findings from the included studies, the methodological quality of the included studies was assessed using a descriptive approach. However, given the narrative and integrative nature of this review and the significant heterogeneity in objectives, designs, and outcomes across studies, formal quantitative scoring and meta-analysis were not performed. Instead, this appraisal was used to contextualize the strength of the evidence, identify common methodological strengths and limitations across the literature, and inform the interpretation of the findings. The outcomes of this assessment are presented in Section 3.5.

### 2.5. Data Synthesis

For each included study, relevant data were extracted, analyzed, and registered into a standardized Excel spreadsheet, recording the following information: (i) author and year of publication; (ii) country of origin; (iii) combat sport; (iv) study design (experimental, cross-sectional, prospective, or retrospective); (v) sample: total number of participants, mean age, competitive level, and sex; (vi) biomechanical focus: stance mechanics, hip rotation, kinematic and kinetic variable; (vii) injury outcomes: injury type, prevalence, and mechanism; (viii) measurement tools; (ix) key findings.

## 3. Results

**Figure 1:**
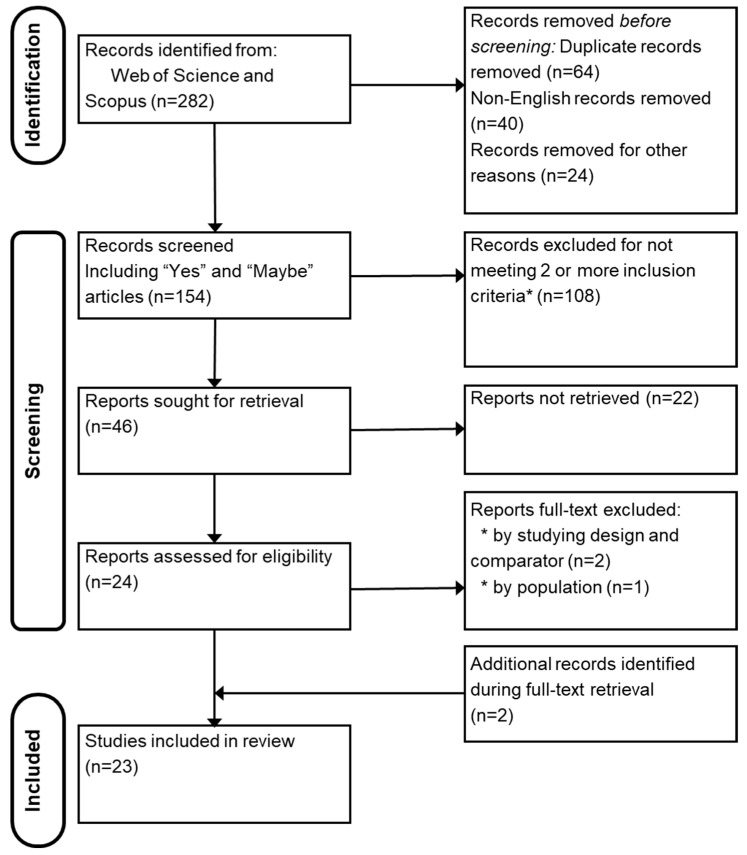
Flowchart of the review process. * Inclusion/Exclusion criteria described in the Materials and Methods section.

The initial search of the Web of Science and Scopus databases identified 282 records. After removing 61 duplicates, 40 non-English articles, and 24 records unrelated to CS or biomechanics, 154 records were retained for screening. Following title and abstract screening, 108 records were excluded for not meeting the inclusion criteria, leaving 46 reports sought for full-text retrieval. Of these, 24 reports were successfully retrieved, and 3 were excluded after full-text review (2 due to study design/comparator, 1 due to population not relevant). In addition to the database search, two relevant studies were identified through a forward manual searching of reference lists and included, as they matched the eligibility criteria previously described in Section 2.1. This resulted in a total of 23 studies included in the review. The study selection process is illustrated in the flowchart (Figure 1).

### 3.1. Characteristics of the Studies

The 23 included studies span Asia, Europe, North America, South America, and Oceania. Countries represented include Korea [7,17,19,25], Spain [8], the USA [4,15,26], Poland [27], Taiwan [7], Iran [28], Italy [5], the Czech Republic [6], Switzerland [20], Canada [3], Brazil [29], Australia [18], Ukraine [30], Romania [31], United Kingdom [32], Turkey [33], Australia [34], and multinational/international championship contexts [21]. Table 1 shows the summary of characteristics, effects, and variables analyzed in each selected study.

In terms of study design, experimental biomechanical studies were the most frequent (n = 12) [5,6,7,8,16,25,26,27,28,29,31,32], while prospective or retrospective cohort designs were reported in five studies [3,4,15,17,21]. The remaining four employed cross-sectional or survey-based designs [18,19,20,30].

The vast majority of the 23 included studies fall into one of two distinct categories: controlled laboratory investigations of biomechanical efficiency (n = 13 experimental studies) or field-based observational reports of injury epidemiology (n = 12 prospective, retrospective, or cross-sectional studies). Notably, there are missing studies concurrently and prospectively measuring high-fidelity biomechanical variables (e.g., joint kinetics during a kick) and directly observing resultant injuries within the same cohort.

### 3.2. Sample Characteristics

Sample sizes ranged from laboratory-based cohorts (≈8–31 athletes) in biomechanics experiments [5,6,7,8,16,25,27,28,29,31,32] to large epidemiological surveillance datasets (hundreds of athlete exposures) [3,4,15,21]. Most biomechanical studies recruited competitive or elite athletes, particularly in Taekwondo [5,6,7,8,16,25,27,32], whereas epidemiological cohorts covered collegiate, national, and world-level competitions [15,17,21] as well as professional MMA/UFC events [4,34] and amateur Muay Thai/K-1/Kickboxing populations [18,20,29].

Sex distribution was male-dominant, though several studies included mixed-sex samples [3,5,15,20]. Age ranged from children/adolescents (e.g., Karate youth cohorts) [3,5] to adult amateurs and professionals in Muay Thai/K-1 [18,20,29,33,34] and collegiate/elite Taekwondo [6,8,15,16,17,25,32]. Across studies, common measurement approaches included 3D motion capture and force plates for mechanics [6,7,8,16,32], pressure platforms [31], impact sensors/dynamometry [8,26,27,29], isokinetic/balance testing [25], and medical/surveillance records or questionnaires for injury outcomes [3,4,15,17,18,19,21].

### 3.3. Mechanical Efficiency

Biomechanical studies consistently highlight the role of stance, hip rotation, and striking surface as the principal determinants of the mechanical efficiency of leg kicks across combat sports [7,8,29,31]. Objective measures, such as joint torques, ground-reaction forces, and postural control, suggest that efficient kicks require precise integration of balance and timing [28]. Athletes with superior postural control demonstrated greater accuracy and stability during unipedal stance [28].

Anthropometric characteristics also influenced performance outcomes, with longer limb segments and greater muscle mass generally associated with higher impact forces; however, intersegmental coordination remained the primary determinant of mechanical efficiency [27].

Overall, mechanical efficiency results from the coordination of stance mechanics, hip rotation, and striking surface choice. Stance sets the base for force transfer, hip rotation provides the primary engine of acceleration, and striking surface selection modulates the balance between power and speed [5,6,7,8,16,27,28,31].

### 3.4. Injury Outcomes

#### 3.4.1. Anatomical Distribution

Injury data suggest that the lower extremities are the most commonly affected zones in combat sports [34]. The knee, ankle, shin, and foot are primary locations of injury, with the patterns influenced by the striking surface, stance mechanics, competitive context, and discipline. In Taekwondo, ankle sprains and foot fractures were frequently linked to instep kicking [8,15]. In shin-dominant striking disciplines, such as Muay Thai and kickboxing, tibial stress injuries were more common [18,20,33]. Additional injuries reported in Muay Thai included epistaxis, concussion, rib trauma, and extremity soft tissue strain.

#### 3.4.2. Incidence and Time-Loss Injuries

Prospective cohort studies in Taekwondo and Karate reported injury incidences ranging from 6–12 injuries per 1000 athlete exposures [15,21]. Surveys of MMA and Muay Thai competitors indicate that 39% of athletes reported at least one health problem, mostly superficial injuries (42.2%) and pain without specific tissue damage [34]. Observational studies during competition found that 91.4% of athletes experienced no health issues [33]. Time-loss injuries, defined as those preventing participation for one or more days, accounted for a substantial proportion of recorded cases, particularly at elite-level tournaments. In the World Karate Championships, approximately 10% of all reported injuries were classified as time loss, with fractures and joint sprains being the most disabling [21].

#### 3.4.3. Mechanisms of Injury

Mechanistically, injuries arose both from attacking actions (e.g., excessive torsional stress on the support leg during pivoting) and receiving actions (e.g., blocked low kicks causing tibial or metatarsal trauma). Overuse injuries were reported less frequently and associated with high training loads, whereas acute traumatic injuries predominated during competition [3,15,21]. Training-related injuries were more often linked to cumulative workload and fatigue [17,29].

#### 3.4.4. Risk Factors

Several studies identified risk modifiers that influenced injury occurrence. Higher acute-to-chronic workload ratios predicted lower-limb injury risk in Taekwondo [17]. Fatigue and autonomic imbalance, measured via heart rate variability in Muay Thai, were associated with greater susceptibility to injury [29]. Psychological and motivational factors also appeared relevant; athletes with higher competitive drive reported greater injury prevalence in Muay Thai, K-1, and Kickboxing [20].

### 3.5. Methodological Quality of Included Studies

The descriptive quality assessment revealed a pattern of methodological characteristics aligned with the two main research streams: controlled laboratory experiments and observational/epidemiological studies.

Laboratory-based biomechanical studies (n = 12) consistently demonstrated high internal validity for measuring discrete kinematic and kinetic parameters. The principal strengths were the use of objective and high-precision instrumentation (e.g., 3D motion capture, force plates) and standardized protocols. In this fragment, the main common limitations were small sample sizes (n < 30, often homogeneous in skill level), which reduced statistical power and generalizability; a focus on single-sport cohorts (predominantly Taekwondo); and the ecological validity of tasks, which may not fully replicate the cognitive and tactical demands of competition.

Observational injury studies (n = 11) provided valuable data on incidence and mechanisms in real-world settings. Prospective designs with active surveillance at competitions offered the most reliable injury data. Common limitations included: variability in injury definitions and reporting methods across studies, which complicates comparison; potential for recall and selection bias in survey-based and retrospective designs; and, in many cases, a lack of adjustment for key confounders such as training load, prior injury history, or protective equipment use.

Notably, only a minority of studies integrated biomechanical and injury outcomes within the same research framework. This methodological divide underscores the core challenge addressed by this review: synthesizing evidence from high-control/low-ecology experiments with low-control/high-ecology observational data to form a coherent understanding of the performance–injury relationship.

The substantial heterogeneity in outcome measures and data collection methodologies across studies is a notable challenge in synthesizing the evidence, limiting direct comparability. To clarify this heterogeneity, the biomechanical and clinical measures employed in the included literature are categorized and presented, along with their primary limitations, in Supplementary Table S1.

## 4. Discussion

This review included 23 studies spanning Taekwondo, Karate, Muay Thai/K-1/Kickboxing, and MMA, integrating biomechanical and injury-related evidence on leg kicks. Across the studies, three technical determinants consistently emerged as primary drivers of mechanical efficiency: stance/support leg mechanics, hip rotation/sequencing, and striking surface selection [5,6,7,8,16,27,28,31]. Injury surveillance consistently identified the lower extremity—particularly the ankle, knee, shin, and foot—as the most frequent and consequential sites of injury in striking contexts [3,4,8,15,17,18,20,21,29]. When these streams are combined, a pattern emerges: the same technical features that enhance efficiency also create load concentrations and tissue-specific injury risk.

### 4.1. Biomechanical Paradox

The core finding of this review is that mechanical efficiency and injury risk are not independent variables; rather, the technical features that optimize impact force are the same features that lead to important loads on musculoskeletal tissues.

#### 4.1.1. Stance Mechanics and the Pivot “Penalty”

Stance emerged as a key determinant of force generation, transfer, and stability. Orthogonal or pivoted support leg position, like in Taekwondo, facilitated greater hip rotation and joint power, improving impact potential, whereas non-pivoted or narrower stances limited force transfer and balance [6,8,16]. However, this mechanical advantage comes with a “penalty.” Pivoted stances increase rotational stress on the knee and tibia of the supporting leg, aligning with ACL-relevant loading. This explains the high incidence of ACL and meniscal injuries found in surveillance data [13,15,19]. Mechanical efficiency in this context is a trade-off between force transfer and joint preservation.

#### 4.1.2. Hip Rotation and Force–Risk Trade-Offs

In general, the hip acts as the biomechanical “engine” of leg kicks. Higher pelvic/hip angular velocity improved to peak impact force and execution speed when the timing along the thigh–shank–foot chain was maintained [7,8]. In fact, proximal-to-distal sequencing—i.e., rotational momentum generated proximally and released distally—was confirmed as critical for efficient impulse transfer, with longer approach distances allowing greater hip acceleration but slower time to contact [7,8,16,32]. However, rapid pelvic acceleration propagated higher rotational and shear loads through the kinetic chain, increasing stress concentrations at the support knee and distal striking surface [8,16]. This suggests that athletes who maximize hip torque for performance may be placing their joints under repetitive high-strain cycles that lead to chronic stress and fatigue-related injuries noted in elite cohorts.

### 4.2. Contact Mechanics and Striking Surface

The choice of striking surface represents the most direct performance–risk compromise identified in the literature. Each strike-surface choice, therefore, reflects a biomechanical compromise between efficiency and injury exposure.

#### Force-Speed Trade-Off

Force-oriented disciplines, such as Muay Thai, prefer the shin as a striking surface [5,8,27,32] because it provides higher impulse transfer and stiffer contact mechanics, maximizing impact force [5,27]. Nevertheless, shin contact enhances the onset of tibial contusions/stress injuries [18,20], periosteal stress, and quadriceps bruising [4,18,20]. Conversely, speed-oriented disciplines (e.g., Taekwondo) favorize instep strikes as they allow for increased strike velocity and scoring speed at the cost of lower force [8,32]. While this improves scoring potential, the smaller bone structures and higher distal velocities lead to the high risk of fractures and sprains documented in these populations [3,5,8,15,18,20,27].

### 4.3. Modifiers: Anthropometry, Fatigue, and Psychology

These trade-offs can be modulated by the individual characteristics of the athlete. Indeed, in the actualization of this potential, individual and psychosocial factors play their decisive role [35]. An athlete’s motivational drive and cultural framing act as a gain control, amplifying or attenuating the consistent application of high-risk, high-efficiency techniques in training and competition, thereby directly influencing injury epidemiology [36].

#### 4.3.1. Anthropometry

While longer limbs or greater lean mass can result in a larger moment arm for higher impact forces [25], this can also mean a greater moment force is applied to the joints at a given instant, as when the kick is blocked. Additionally, coordination/timing performance relies deeply on the morphology of the individual [27]. This could suggest that the overall anthropometry of the athlete determines the speed and force produced by the subject.

#### 4.3.2. Fatigue

Fatigue can influence the transition from an efficient strike to an injurious one. With the alteration of the balance between agonist and antagonist muscles and the consequent decline in neuromuscular control [28] due to fatigue, kicking efficiency is reduced, and thus even a technically correct kick could become a high-risk event [17,29]. In other words, the neuromuscular system’s ability to reduce the stress on the joints through proper muscle coactivation is compromised. This is more risky in young cohorts, as less mature neuromuscular control is particularly vulnerable to imbalance between performance goals and tissue tolerance [3,25].

#### 4.3.3. Perceived Versus Actual Risk

As studies have shown [18], injury is often viewed as an inherent component of combat sports. This makes the athletes prioritize performance (e.g., impact force or speed) over joint preservation. While athletes might perceive the injuries of the striking surface as the primary threat—leading to a psychological focus on shin conditioning—they often lack a corresponding concern for the support leg’s ligamentous safety [18]. This creates a false perception of the athlete where the most dangerous mechanics are the least feared.

Moreover, evidence from Muay Thai and Kickboxing [20] indicates that high competitive drive and motivational orientation are significant predictors of injury prevalence. Athletes with higher drive tend to misinterpret or ignore physiological signals of fatigue and autonomic imbalance [20]. This drive–risk relationship might suggest that the psychological commitment to efficiency often overrides the biological warning systems intended to prevent tissue failure.

#### 4.3.4. Gender Dimorphism

Anatomical, hormonal, and neuromuscular dimorphisms suggest plausible ways in which the proposed efficiency–injury model components might be differentially expressed or modulated in female athletes. The typically wider quadriceps angle (Q-angle) in females alters the alignment of the patellofemoral joint and the force vector of the quadriceps on the tibia [37]. During a pivoted kick, this could theoretically increase lateral patellar shear and exacerbate the rotational stress on the supporting ACL [38,39]. Also, fluctuations in estrogen across the menstrual cycle may change ligament laxity and collagen synthesis [40,41], lowering the load threshold at which ligaments succumb to the torsional and shear forces generated during the kicks.

### 4.4. Practical Implications

The synthesis of biomechanical efficiency, injury epidemiology, and psychological risk factors suggests that performance and injury prevention in combat sports are linked. Hence, the following targeted interventions are proposed.

#### 4.4.1. For Coaches and Technical Staff

Coaches should recognize the stance/pivot high-performance/risk mechanics [6,8,16,17]. Hence, coaches should treat it as a specialized skill. Technical practice should therefore emphasize controlled pivot execution and progressive loading of support leg stability to minimize torsional knee stress, for example, practice kicks starting with a minimal pivot (e.g., 30°), focusing on stability, then gradually increasing the rotation angle as control improves.

Moreover, coaches should train athletes to match their striking surface to the discipline context. For instance, using the shin for high-power, low-speed delivery (Muay Thai/MMA) requires specific bone-loading conditioning, whereas instep strikes in scoring-based sports (Taekwondo/Karate) require specific ankle stabilization exercises to prevent fractures [3,15,18,20,21]. Coaches should actively target supplemental training based on the discipline’s dominant risks. If relying on pivoted kicks, prioritizing single-leg stability, Bulgarian split squat [42], eccentric hamstring strength (e.g., Nordic curls), and Romanian deadlift [43] in gym work can protect the support knee [44]. Focusing on ankle strengthening (e.g., banded eversions) and foot arch exercises (short-foot drills, marble pickups) for strikes with the instep is also beneficial.

Technical staff should work to decouple the normalization of risk from high performance by recognizing that a very motivated athlete [20] is likely to hide fatigue and pain. Hence, coaches should use objective measures (e.g., kick speed drop-off or heart rate variability) rather than athlete self-reporting to determine the training volume.

#### 4.4.2. For Athletes

Athletes should be educated on the performance–risk trade-offs of technical choices. Developing bilateral proficiency in stance and striking surface may diversify tactical options and distribute loading more evenly across limbs. They should actively engage in targeted supplemental training based on their discipline’s dominant risks. Workload monitoring, including acute-to-chronic training ratios, should be adopted to reduce overuse and fatigue-related injuries [17,29]. Regular balance and proprioceptive training may mitigate the instability that predisposes athletes to support leg injuries [25,28].

#### 4.4.3. For Clinicians and Sports Medicine Practitioners

Clinical screening for strength and balance asymmetries (e.g., between dominant and non-dominant legs) can identify athletes at elevated risk [25]. Rehabilitation should address both the injured tissue and the mechanical chain (e.g., retraining hip control after ankle injury, or support leg stability after knee injury). Injury surveillance systems in competition and training should distinguish between instep, shin, and knee-related injuries, enabling more precise risk monitoring [3,4,15,18,20,21].

When treating an injured combat athlete, clinicians should assess their “Competitive Drive” [20]. High-drive athletes may require more rigid return-to-play protocols to prevent them from returning too early due to an underestimated perception of risk.

### 4.5. Future Directions

While the current literature mentions the basic knowledge of leg kicks, several critical gaps remain. Future research should prioritize the following four areas to move the field toward a more predictive and preventive science.

The majority of studies recruited predominantly male participants, with limited attention to female athletes or youth cohorts. Future work should conduct sex-disaggregated analyses of biomechanical efficiency and injury risk, as sex-related differences in joint laxity, neuromuscular control, and injury profiles are well documented in other sports [38]. Similarly, research should focus on adolescent development phases, where imbalances in growth, coordination, and strength may exacerbate injury vulnerability [3,25].

Training load ratios are linked to injury outcomes [17,29], yet most studies were short-term or cross-sectional. Future research should employ longitudinal cohort designs that track athletes across training cycles and competitive seasons, enabling clearer identification of workload thresholds associated with injury onset. Integration of injury surveillance with biomechanical testing would allow direct mapping of mechanical efficiency metrics onto long-term health outcomes.

Research is needed to evaluate the effectiveness of prevention strategies—for example, proprioceptive training, stance-modification drills, and tibial/ankle conditioning—on both reducing injury incidence and maintaining performance outcomes. Such interventions should be tested in collaboration with coaches, clinicians, and athletes, ensuring feasibility in real-world training contexts.

Although this review included Taekwondo, Karate, Muay Thai, Kickboxing, and MMA, the evidence base remains skewed toward Taekwondo. Future studies should expand direct biomechanical comparisons across sports. Such cross-sport analyses could clarify how rule sets and coaching traditions shape the efficiency–injury continuum.

Advances in wearable sensors, inertial measurement units (IMUs) [45], and machine learning analytics [46,47] provide opportunities for more ecological measurements [48,49,50]. Future studies should leverage these technologies to validate laboratory findings in ecological, training, and competitive environments. This would support data-driven feedback systems for both performance enhancement and injury prevention.

A link between competitive drive, fatigue, and injury was found [18,20]. Future research should adopt a multivariate approach. Integrating biomechanical metrics (e.g., peak hip velocity) with psychological assessments (e.g., risk perception scales) and physiological data (e.g., Heart Rate Variability) would allow researchers to create a “Risk Profile” for individual athletes. This could lead to machine learning models [46,47] capable of predicting injury windows based on the interaction of technical output and psychological state.

Ultimately, the goal should be to develop predictive, quantitative models that can estimate injury probability for a given athlete based on the weighted inputs of their biomechanical technique, physiological readiness, and psychosocial profile (e.g., score on risk-taking propensity scales) [51]. This would move the field from descriptive frameworks to actionable, personalized risk assessment.

Few controlled experiments directly compared shin vs. instep striking, or pivot vs. non-pivot stances, under standardized conditions. Randomized, lab-based trials examining these variations could establish causal links between technique, mechanical efficiency, and injury mechanisms, strengthening the biomechanical–epidemiological synthesis.

Beyond combat sports, these findings contribute to broader sports biomechanics by illustrating how technique sequencing interacts with injury mechanisms in dynamic striking tasks. The efficiency–injury trade-off model may therefore inform not only martial arts training but also sports medicine approaches to activities with similar rotational loading, such as soccer kicking [52], tennis [53,54,55], or hockey shooting [56,57].

### 4.6. Strengths and Limitations

The principal strength of this review lies in its cross-disciplinary synthesis. By integrating biomechanical and injury evidence on leg kicks, this review provides a unified framework that moves beyond the typical “lab vs. clinic” divide. Hence, this review highlights the direct overlap between performance determinants and injury mechanisms, offering a unified framework relevant to athletes, coaches, and clinicians. Previous research streams have tended to isolate either mechanical efficiency (laboratory biomechanics) or injury incidence (epidemiology and surveillance).

The inclusion of diverse CS (Taekwondo, Karate, Muay Thai, Kickboxing, MMA) further strengthens the generalizability of the findings across striking-based disciplines. However, discipline distribution was uneven, with Taekwondo being disproportionately represented relative to other sports, such as Muay Thai and MMA, which are underrepresented. This gives more weight to instep-dominant sports, potentially biasing the synthesis toward rule sets emphasizing speed and scoring efficiency. This limitation may bias the outcomes; therefore, future studies should consider conducting larger, separate analyses by discipline.

Several other limitations should temper interpretation. First, sex representation was uneven, with the majority of included participants being male. Only a minority of studies provided sex-disaggregated analyses, limiting the applicability of findings to female athletes. Second, sample sizes in biomechanics experiments were generally small (often fewer than 20 participants), which constrains statistical power and the representativeness of mechanical efficiency findings. Third, heterogeneity in measurement approaches—including motion capture systems, force sensors, and injury surveillance protocols—limited direct comparability of outcomes across studies. The narrative and integrative nature of this review, necessitated by the heterogeneity in designs, populations, and measures described above, means that its findings should be interpreted as hypothesis-generating rather than as definitive conclusions. Furthermore, because current data are mostly based on tests performed in the laboratory, there is a need for future longitudinal studies that collect data in real-time sparring environments using wearable sensors (e.g., IMUs) and video systems. Taking care that the protocol of using wearable sensors must be followed in practical exercises on a single player, avoiding contact with another participant.

Finally, the main search relied solely on the Web of Science Core Collection and Scopus, excluding relevant literature indexed in other sources (e.g., PubMed, SPORTDiscus), which substantially increased the risk of publication bias and limited the comprehensiveness of the included evidence. In addition, no manual backward or forward reference searching was systematically performed beyond these sources, and it may have excluded relevant studies indexed elsewhere.

## 5. Conclusions

This review proposes and synthesizes evidence supporting the concept that mechanical efficiency and injury risk in leg kicks across combat sports are inseparable. The technical determinants of a powerful strike—specifically the pivoted stance and proximal-to-distal hip sequencing—appear to be closely associated with the same mechanisms that generate hazardous loads on the support leg knee and striking surface. Pivoted stances and rapid hip rotation enhance impact force and timing but elevate knee loading; shin strikes maximize impulse transfer but predispose athletes to tibial trauma; and instep strikes facilitate speed yet expose the foot and ankle to fractures and sprains.

By connecting laboratory biomechanics with epidemiological evidence, this review constructs a performance–safety continuum that offers a plausible explanatory framework for why efficiency gains frequently coincide with injury vulnerabilities. The framework suggests practical guidance for athletes, coaches, and clinicians: technical refinement, load monitoring, and targeted conditioning can balance the trade-offs between maximizing performance and minimizing risk. However, considering the connections between kinematic parameters, joint and tissue stress are drawn from a fragmented evidence base where biomechanical cause and injury effect are rarely measured in conjunction.

## Figures and Tables

**Table 1:** Characteristics and effects of studies about mechanical efficiency and injury risk in leg kicks across combat sports.

**Study**	**Country**	**Combat Sport**	**Study Design**	**Sample**	**Biomechanical Focus**	**Injury Outcomes**	**Tools Used**	**Key Findings**
Ramakrishnan et al., 2018 [26]	USA	Taekwondo	Experimental	n = 10 not specified	Kinetic variable: Energy transfer measurement	None	Force sensors	New method based on the force–time relation to quantify kick energy.
Park et al., 2021 [19]	Korea	Mixed Combat sports	Cross-sectional	n = 160 (m)	None	Injury prevalence (age)	Questionnaire	Injury risk increased with age, especially for lower limb injuries.
Zhao et al., 2022 [15]	USA	Taekwondo	Prospective	n = 183 m: 132 f: 41	None	Injury prevalence	Injury forms	In taekwondo, the lower limb is the most affected with high rates of ankle sprains and foot fractures.
Podrigalo et al., 2017 [30]	Ukraine	Martial Arts	Cross-sectional	n = 28 m: 20 f: 8	Hip rotation: Joint goniometry	None	Goniometer	Hip flexibility asymmetries are linked to kick efficiency.
Hsieh et al., 2012 [7]	Taiwan	Taekwondo	Experimental	n = 10 not specified	Hip rotation in a roundhouse kick	None	Motion capture	High hip rotation increases impact force and speed.
Kim et al., 2022 [16]	Korea	Taekwondo	Experimental	n = 20 m: 10 f: 10	Stance mechanics: ACL risk during pivot	Injury mechanism: risk modeling	Motion analysis	The pivoting leg shows high ACL stress in the supporting leg.
Daniel & Razvan, 2014 [31]	Romania	Karate	Experimental	n = 12 m: 10 f: 2	Stance mechanics: Plantar pressure vs. striking	None	Pressure plates	Higher plantar pressure correlated with stronger strikes.
Gorski & Orysiak, 2019 [27]	Poland	Taekwondo	Experimental	n = 16 (m)	Stance mechanics: Limb dominance in kicks	None	Force plates	Dominant leg kicking produced greater impact force.
Negahban et al., 2013 [28]	Iran	Taekwondo	Cross-sectional	n = 28 (m)	Stance mechanics: Postural control	None	Force plate	Taekwondo experts had better balance compared to shooting.
Everard et al., 2025 [32]	UK	Taekwondo	Prospective	n = 20 not specified	Stance mechanics	None	Motion capture	Stimulus cues improved performance, increasing speed and accuracy.
Robbiani & Filippi, 2025 [20]	Switzerland	Muay Thai, K-1, Kickboxing	Cross-sectional	n = 325 m: 244 f: 81	None	Injury prevalence	Surveys	High motivation is linked with a higher injury rate.
Falco et al., 2009 [8]	Spain	Taekwondo	Experimental	n = 16 m: 12 f: 4	Kinetic: Kick distance	None	Force plates	A longer distance increased the force but slowed execution.
Lee et al., 2025 [17]	Korea	Taekwondo	Prospective	n = 98 m: 72 f: 26	None	Injury type	Monitoring logs	A high workload ratio increases injury incidence
Dong et al., 2025 [25]	Korea	Taekwondo	Cross-sectional	n = 40 m: 25 f: 15	kinetic	Injury prevalence	Isokinetic dynamometer and surveys	Muscle imbalance is linked to higher injury risk.
Zetaruk et al., 2000 [3]	USA	Karate	Retrospective	n = 263 m: 169 f: 94	None	Injury prevalence	Surveys	Most injuries were located in the lower limb, sprains being the most common.
Fares et al., 2019 [4]	USA	UFC (MMA)	Retrospective	n = 285 m: 249 f: 36	None	Injury prevalence and mechanisms	Medical records	Head injuries are the most common type of injury, followed by upper limb and lower limb injuries.
Strotmeyer & Lystad, 2017 [18]	Australia	Muay Thai	Cross-sectional	n = 136 m: 96 f: 40	None	Perceived risk	Surveys	Muay Thai fighters underestimate the risk of injury relative to other contact sports based on their own ability to negotiate risk.
Junior et al., 2025 [29]	Brazil	Muay Thai	Cross-sectional	n = 21 (m)	Kinetic	None	Heart rate variability monitor, Frequency speed of kick test	Heart rate variability, as an injury risk indicator, can be used by coaches to prevent injuries.
Casolino et al., 2012 [5]	Italy	Taekwondo (Youth)	Cross-sectional	n = 24 not specified	Kinematic	None	Video analysis	Young athletes tend to use simpler techniques in competition, suggesting that coaches should emphasize coordination exercises to enhance performance.
Jandačka et al., 2013 [6]	Czechia	Taekwondo	Cross-sectional	n = 12 m: 6 f: 6	Stance mechanics	None	Force plates, movement capture analysis	The stance has little effect on kicking speed but a strong effect on hip power, which should be taken into account during training.
Augustovicova et al., 2019 [21]	International	Karate	Prospective	n = 778 m: 558 f: 220	None	Injury prevalence	*World Karate Federation* surveillance system	Only 10% injuries caused time loss, mainly due to upper-extremity fractures.
Doherty et al., 2025 [34]	Australia	MMA/Muay Thai	Prospective	n = 238 m: 180 f: 58	None	Injury prevalence	Questionnaire	High incidence of illness and injury during and after competition, with effects mainly limited to the 17 days post-competition.
Hallaçeli et al., 2025 [33]	Turkey	Muay Thai	Prospective	n = 663 m: 445 f: 218	None	Injury prevalence: Epidemiological analysis of athlete injuries	Direct Clinical Observation	Injury rates in official Muay Thai competition were low, likely due to medical supervision, protective equipment, and timely Referee Stop Contest (RSC) decisions, which help limit injury severity.

n: number of participants. m: number of male participants. f: number of female participants. TKD: taekwondo. ACL: anterior cruciate ligament. MMA: mixed martial arts. UFC: Ultimate Fighting Championship. Regarding combat sports, the majority of studies focused on Taekwondo (n = 12) [5,6,7,8,15,16,17,25,26,27,28,32], followed by Karate (n = 3) [3,21,31], Muay Thai/Kickboxing/K-1 (n = 5) [18,20,29,33,34], and Mixed Martial Arts/UFC (n = 4) [4,19,30,34].

## Data Availability

No new data were created or analyzed in this study. Data sharing is not applicable to this article.

## Supplementary Materials

The following supporting information can be downloaded at: https://www.mdpi.com/article/10.3390/healthcare14040430/s1, Table S1: Categorization of outcome measures and their methodological considerations in studies on leg kick biomechanics and injury.

## Abbreviations

The following abbreviations are used in this manuscript:

CS	Combat Sports
ACL	Anterior Cruciate Ligament
MMA	Mixed Martial Arts
DOI	Digital Object Identifier
WKF	World Karate Federation
UFC	Ultimate Fighting Championship
TKD	Taekwondo
