# Supplementary material for: Mechanical Efficiency and Injury Risk in Leg Kicks Across Combat Sports: A Narrative Review of Stance, Hip Rotation, and Striking Surface Effects
Source: Healthcare (Basel). 2026 Feb 9;14(4):430. doi: 10.3390/healthcare14040430 (PMC12940734; doi:10.3390/healthcare14040430)
Supplement: Supplementary file 1 [file healthcare-14-00430-s001.zip › healthcare-4122535-supplementary.pdf]

Supplementary Material

Table S1: Categorization of outcome measures and their methodological considerations in studies on leg kick biomechanics and injury.

Category of Measure	Specific Variables (from included studies)	Typical Instruments / Methods	Key Limitations for Comparability
**Kinematic**	- Foot/limb linear velocity<br>- Joint angular velocity/displacement (hip, knee, ankle)<br>- Execution time (time-to-target)<br>- Proximal-to-distal sequencing timing	- 3D optical motion capture (gold standard)<br>- 2D video analysis<br>- Inertial Measurement Units (IMUs)	- **Marker set & biomechanical model:** Differences in anatomical landmark definitions and segment models affect joint angle calculations.<br>- **Sampling rate & accuracy:** Varies between systems (high-speed cameras vs. standard video).<br>- **Task standardization:** Differences in target type (fixed bag vs. moving target), distance, and instructed effort ("maximal" vs. "technical" kick).
**Kinetic / Impact**	- Impact/peak force<br>- Impulse<br>- Ground reaction force (magnitude, center of pressure)<br>- Joint moments (estimated via inverse dynamics)	- Force plates (for GRF)<br>- Instrumented targets / punching mats with piezoelectric or strain-gauge sensors<br>- Pressure plates<br>- Isokinetic dynamometers (for isolated strength)	- **Sensor calibration & type:** Different sensor technologies (piezoelectric vs. strain gauge) yield different force-time curves.<br>- **Target properties:** Mass, stiffness, and mounting of the target greatly influence measured impact force.<br>- **Lack of standardization:** No common protocol for impact measurement, making absolute values non-comparable across studies.
**Clinical & Epidemiological**	- Injury incidence (per 1000 athlete-exposures)<br>- Injury prevalence (% of athletes)<br>- Injury type (e.g., sprain, fracture, contusion)<br>- Injury mechanism (e.g., pivot, block)<br>- Time-loss duration	- Medical records / tournament logs<br>- Prospective injury surveillance forms (e.g., OSICS)<br>- Retrospective questionnaires / surveys<br>- Clinical examination	- **Injury definition:** Lack of consensus on what constitutes a "reportable injury" (e.g., all complaints vs. only time-loss injuries).<br>- **Reporting bias:** Under-reporting of minor injuries, recall bias in surveys.<br>- **Exposure calculation:** Inconsistent methods for calculating athlete-exposure units (per hour, per session, per match).<br>- **Context:** Data from competitions vs. training environments are not directly comparable.
